# Cognitive Reserve Is Not Associated With Hippocampal Microstructure in Older Adults Without Dementia

**DOI:** 10.3389/fnagi.2019.00380

**Published:** 2020-01-23

**Authors:** Judith Kalzendorf, Katharina Brueggen, Stefan Teipel

**Affiliations:** ^1^DZNE, German Center for Neurodegenerative Diseases, Rostock, Germany; ^2^Department of Psychosomatic Medicine, University Medicine Rostock, Rostock, Germany

**Keywords:** mean diffusivity, hippocampus, cognitive reserve, Alzheimer’s disease, amyloid, diffusion tensor imaging

## Abstract

**Objective:**

Mean Diffusivity (MD) as measured by diffusion tensor imaging (DTI) can be used to detect microstructural alterations of the brain’s gray matter (GM). A previous study found that higher education, which is a proxy for cognitive reserve (CR), was related to decreased hippocampal MD in middle-aged healthy adults, indicating decreased microstructural damage in more educated participants. Based on this study, we aimed at determining the role of hippocampal GM MD in the interaction of AD pathology and CR in older people without dementia.

**Method:**

We used a sample of 52 cognitively normal people and 38 participants with late mild cognitive impairment (LMCI) from the ADNI database. MCI and cognitively normal participants were analyzed separately. Using linear models, we regressed hippocampal GM MD on CR (quantified by a composite score), amyloid status and the interaction of both, adjusting for age, gender and memory score.

**Results:**

CR was not associated with hippocampal GM MD and hippocampal GM volume. Also, no interaction of amyloid status and CR was found.

**Conclusion:**

Our results do not confirm an association of CR and hippocampal GM MD in older adults. In contrast to previous studies, we did not find an association between CR and microstructural, nor macrostructural alterations of the hippocampus in older adults. More research is needed to determine the influence of CR on hippocampal microstructural integrity in relation to age and AD pathology.

## Introduction

Diffusion tensor imaging (DTI) can be used to quantify microstructural changes in the brain *in vivo*. It has mostly been used to assess white matter (Assaf and Pasternak, 2008), but more recently, it has also been applied to study gray matter (GM) microstructural integrity (Weston et al., 2015). Mean diffusivity (MD), a scalar DTI measure, quantifies the average degree of diffusion of water molecules in all directions (Le Bihan et al., 2001). Evidence from animal models suggests that cell atrophy leads to a loss of microstructural barriers to diffusion such as cell membranes, thus increasing MD in the brain’s gray matter at an early stage of atrophy (Zerbi et al., 2013).

In cognitively healthy older people, increased MD of GM structures has been found to correlate with decreased cognitive function (Carlesimo et al., 2010; Kantarci et al., 2011; den Heijer et al., 2012; Salminen et al., 2016; Gyebnár et al., 2018). Specifically, Kantarci et al. (2011) found that medial temporal lobe MD was negatively related to memory performance in a sample of healthy older people and mild cognitive impairment (MCI) patients. Similarly, left hippocampal MD correlated with memory performance in healthy people older than 50 years (Carlesimo et al., 2010), whereas no association was found in younger people.

The concept of cognitive reserve (CR) was introduced to explain the discrepancy between the level of neuropathology and the level of ante-mortem functional impairment in brain autopsy studies (Katzman et al., 1988). This discrepancy is assumed to depend on lifetime experiences, such as education, occupation, or hobbies. More cognitively challenging lifetime experiences thus protect individuals from functional impairment, either by compensating or by preventing neuropathological lesions. As an example, Bennett et al. (2003) found that the association of neuropathology and cognitive impairment was moderated by years of formal education. More precisely, stronger negative correlations of neuropathology and cognitive function were found in participants with fewer years of education. Thus, the concept of CR represents an active cognitive mechanism that protects from cognitive impairment.

Cognitive reserve needs to be distinguished from the closely related concepts of brain reserve and brain maintenance (Barulli and Stern, 2013). According to the concept of brain reserve, brain measures such as total intracranial volume or head circumference explain the differential susceptibility to cognitive impairment at a given level of neuropathological lesion. In contrast, brain maintenance refers to healthy aging and the mechanisms that protect healthy older people against neuropathology or age-related cognitive deterioration (Barulli and Stern, 2013).

The quantification of CR is still a matter of debate (Nilsson and Lövdén, 2018). Most researchers use single proxies, such as years of education (Meng and D’Arcy, 2012), occupational status or measures of premorbid IQ (Alexander et al., 1997). Other measures include physical or social activities (Scarmeas and Stern, 2003). However, using a single proxy (e.g., years of education) to measure a latent construct (CR) may lead to a biased measurement. Also, proxy variables such as education may affect the risk of dementia in ways that are unrelated to CR. As an example, more years of education are directly related to health status (Silles, 2009). These problems may be resolved by using specific CR questionnaires that were developed to provide a more comprehensive measure of CR (Valenzuela and Sachdev, 2007; Nucci et al., 2012). Another way is the calculation of a measure of the discrepancy between cognitive performance and neuropathology (Reed et al., 2010; van Loenhoud et al., 2017). As long as there is no consensus on the operationalization of CR, a composite score combining several proxies is preferable. A factor analytic approach has been previously used to determine a composite CR score (Scarmeas et al., 2004; Stern et al., 2005; Solé-Padullés et al., 2009). The resulting CR score summarizes shared variance between established proxy measures and contains less proxy-specific variance than a single proxy measure. As our sample (ADNI) is known to be highly educated, we expect to increase variance thereby avoiding a ceiling effect in our CR measure by using a composite score. At the same time, we think that a composite score can capture the concept of CR more precisely by reducing proxy specific variance.

Reviewing the evidence on the association of brain changes and CR, Arenaza-Urquijo et al. (2015) proposed two distinct mechanisms of CR. The *compensatory mechanisms* of CR are assumed to take effect once neuropathology is present in the brain. Reserve would then delay the onset of cognitive symptoms despite the neuropathology. Evidence for this mechanism has been found in studies on patients with Alzheimer’s disease (AD) using multiple indices of AD pathology, such as Aβ42 CSF, hypometabolism and atrophy (Querbes et al., 2009; Solé-Padullés et al., 2009). For example, Solé-Padullés et al. (2009) found a negative association of whole-brain volume and CR in participants with MCI and mild AD at a given level of cognitive performance. The *neuroprotective mechanisms* of CR have mainly been reported in healthy subjects (Landau et al., 2012a), but also in animal and intervention studies (Valenzuela et al., 2003; Costa et al., 2007; Erickson et al., 2011). They refer to direct positive effects of lifestyle factors on neuropathological processes (Arenaza-Urquijo et al., 2013a). Arenaza-Urquijo et al. (2015) postulated a model assuming differential effects of CR according to the stage of AD progression: a neuroprotective effect of CR in cognitively healthy individuals, progressing toward a compensatory effect of CR in individuals at a more advanced stage of the disease.

When it comes to hippocampal integrity and CR, inconsistent results were found so far. On the one hand, in healthy older people with abnormal CSF amyloid, a negative association of CR proxies and hippocampal volume was found, suggesting a *compensatory mechanism* of CR on hippocampal integrity (Arenaza-Urquijo et al., 2013b). However, in another study with healthy older people with low amyloid burden, no association of hippocampal volume and years of education was found, suggesting differential effects according to amyloid status (Arenaza-Urquijo et al., 2013a). On the other hand, evidence for a *protective mechanism* of CR on the hippocampal atrophy rate was found in older healthy individuals (Valenzuela et al., 2008). Additionally, Piras et al. (2011) found lower MD, i.e., better preserved hippocampal microstructural integrity, in better educated cognitively normal middle-aged individuals. This would also indicate a protective mechanism of CR on hippocampal microstructural integrity.

The present study aims at determining the role of hippocampal GM MD in the interaction of AD pathology and CR in older people without dementia.

More specifically, our first aim was to examine the association of CR and hippocampal GM MD in older people with normal cognition (NC) and with MCI after controlling for the level of memory performance. In accordance with the results by Piras et al. (2011), we assumed a negative association of hippocampal CR and GM MD.

Second, following the model by Arenaza-Urquijo et al. (2015), we examined whether the presence of amyloid load moderated the association of CR and hippocampal GM microstructural integrity. We expected a significant interaction of CR and amyloid load in both, MCI and NC subjects. This interaction would be assumed to result from a negative association of a composite CR score and hippocampal MD in amyloid negative participants, indicating a *neuroprotective* mechanism. In amyloid positive participants, we expected a *compensatory* effect of CR, resulting in a positive association of a composite CR score and hippocampal MD.

For comparison, we also evaluated the association of CR and hippocampal volume as a measure of hippocampal *macro*structural integrity.

## Materials and Methods

### ADNI Database

Data used in the preparation of this article were obtained from the ADNI database^1^. The primary goal of ADNI has been to test whether serial MRI, PET, other biological markers, and clinical and neuropsychological assessment can be combined to measure the progression of MCI and early AD. In the present study, data was used from the ADNI2 study, which began in 2011, and from the ADNI3 study, which started in 2016. For up-to-date information, see www.adni-info.org.

Written informed consent was obtained from all study participants according to the Declaration of Helsinki, and Ethical approval for data collection and sharing was given by the institutional review boards of the participating institutions in the ADNI.

### Inclusion and Exclusion Criteria

To be included in the current study, the following measurements had to be available:

- DTI and T1-weighted MRI scans from the same visit,
- American National Adult Reading Test (ANART), years of education and recent occupation,
- Composite memory score (Crane et al., 2012),
- Florbetapir cortical summary measurement, normalized by the cerebellum.

Because of the variability of DTI data across study sites (Teipel et al., 2012), we only included sites in our dataset that contributed at least three participants.

We included 42 cognitively NC participants and 52 participants with a diagnosis of late MCI (LMCI). Detailed inclusion and exclusion criteria for the ADNI study can be found at adni.loni.usc.edu. In brief, the diagnostic criteria for NC are: MMSE scores between 24 and 30; a Clinical Dementia Rating score of 0; no depression; no MCI; no dementia. LMCI was diagnosed with MMSE scores between 24 and 30; memory impairment as determined by an education adjusted threshold of the Logical Memory II subtest of the Wechsler Memory Scale – Revised; subjective memory concern reported by subject, informant or clinician; a CDR of 0.5; absence of significant levels of impairment in other cognitive domains; essentially preserved activities of daily living; absence of dementia. In contrast to early MCI, LMCI was diagnosed if the performance in the Logical Memory Test fell under a lower education adjusted threshold.

We chose LMCI patients as our MCI sample because the degree of AD pathology in the early MCI sample is not different from that in healthy controls (Grothe et al., 2014; Lee et al., 2017).

### Cognitive Measures

#### Memory Score

We used the memory composite from the ADNI database as a comprehensive measure of memory performance. This composite score resulted from a factor analytic approach applied on the verbal episodic memory scores available in ADNI (Crane et al., 2012).

#### Cognitive Reserve Score

A composite CR score was obtained using factor analysis [R package *Psych* (Revelle, 2018)] on established proxy variables of CR: recent occupation, years of education, premorbid verbal IQ. Premorbid verbal IQ was measured using the American National Adult Reading Test (ANART). In the ANART, participants have to pronounce irregular English words and the number of mispronounced words is recorded. Occupation was classified into three levels (professional/managerial; skilled; partly skilled) as described in Lo and Jagust (2013). As the resulting variable “occupation” was not continuous, the factor analysis was computed on a mixed correlation matrix, also including polychoric correlations. The numbers of factors to extract was set to one. We inverted the resulting CR score so that it was positively correlated to years of education for easier interpretation.

The three proxy variables were included in the factor analysis. The resulting CR factor explained 55 % of the common variance in the three variables across all cases. More information on the results of the factor analysis can be found in the Supplementary Table S1.

### Neuroimaging

#### Image Acquisition

All ADNI2 participants were scanned with a 3 Tesla GE Medical Systems scanner. T1-weighted scans were obtained using a spoiled gradient echo sequence (SPGR; 256 × 256 matrix; voxel size = 1.2 × 1.0 × 1.0 mm^3^). DTI scans were obtained using an echo planar spin echo sequence (EP/SE) with 5 B0 images and 41 gradient directions (more details can be found at https://adni.loni.usc.edu/wp-content/uploads/2010/05/ADNI2_GE_3T_22.0_T2.pdf).

Six LMCI participants were scanned according to the ADNI3 protocol. Three of them were scanned with a 3T SIEMENS scanner and therefore, their T1-weighted scans were acquired using a magnetization prepared rapid gradient-echo sequence (MPRAGE). T1-weighted images of the other three ADNI3 participants were scanned with a GE systems scanner using an SPGR sequence (256 × 256 matrix; voxel size = 1.2 × 1.0 × 1.0 mm^3^). DTI scans were obtained following the ADNI3 basic DTI protocol with a single *b* = 1000 s/mm^2^ and 48 gradient directions (more information about the ADNI3 MRI protocols can be found at http://adni.loni.usc.edu/wp- content/uploads/2017/07/ADNI3-MRI-protocols.pdf). Due to the different imaging acquisition protocols, study phase (ADNI2/ADNI3) was included as a covariate in the analysis of LMCI data.

18F-Florbetapir-PET data were acquired on multiple instruments of varying resolution and following different platform-specific acquisition protocols. The acquisition delay between PET and MRI was on average 73 days (SD = 136 days, range 0–734). The cutoff to determine amyloid positivity (Aβ+) was 1.11 for the summary standardized uptake value ratio (SUVR) normalized by the whole cerebellum reference region, as has been previously validated in the ADNI data set (Landau et al., 2012b)^2^.

#### Processing of MRI and DTI Data

Deformation-based analysis of the T1-weighted scans was performed using SPM8 (Wellcome Trust Centre for Neuroimaging, London, United Kingdom)^3^ implemented in Matlab 2013b (Mathworks, Natwick). First, MRI scans were segmented into GM, WM, and CSF partitions using the VBM8 toolbox. Then, the images were normalized into the Montreal Neurological Institute (MNI) reference space using the Diffeomorphic Anatomical Registration Through Exponentiated Lie (DARTEL) algebra algorithm with modulation for non-linear transformation components only.

Diffusion tensor imaging data were preprocessed using the diffusion toolbox of FMRIB Software Library (FSL) correcting for eddy currents and head motion. Then, skull stripping was performed using the Brain Extraction Tool. Diffusion tensors were fitted with DTIfit and the resulting MD maps were coregistered to the T1-weighted scans.

When examining GM MD in neurodegenerative diseases and in older people, partial volume effects (PVE) may arise due to contamination by cerebrospinal fluid and inflate the MD measure. A CSF contamination model was applied to correct for PVE, as described in Henf et al. (2018). The coregistered and partial volume-corrected MD maps were normalized to MNI space by applying the deformation fields obtained for the T1-weighted scans.

#### Extraction of Hippocampal Gray Matter Mean Diffusivity and Volume

Individual GM masks were applied, so that only voxels with a GM probability of more than 50% were included. The Harvard-Oxford structural atlas (Desikan et al., 2006) was used to extract hippocampal MD and volume values for each subject from the normalized data.

### Statistical Modeling

SPSS 23 was used for all statistical analyses. For the comparisons of the groups, we calculated student *t*-tests or Mann-Whitney-*U*-tests as *post hoc* pairwise comparisons (*p* < 0.05), depending on the normality of the data distribution.

Prior to estimating multiple regression models, we performed regression diagnostics to verify the statistical assumptions of linear regression (Williams et al., 2013; Eid et al., 2017). To determine the association of CR and hippocampal GM MD or volume within each group (NC, LMCI), we estimated multiple regression models, adjusted for age, gender and memory performance. Study phase (ADNI2/ADNI3) was an additional covariate in the LMCI subgroup.

Next, we examined whether amyloid load moderated the association between CR and hippocampal microstructural and macrostructural integrity within the diagnostic groups. We estimated multiple regression models including the predictors CR score, amyloid status (Aβ+ vs. Aβ−), and the interaction term amyloid status^∗^CR, with the nuisance variables age, gender, the composite memory score and study phase. For each regression analysis, we performed a *post hoc* power analysis using the software G^∗^Power (Version 3.1.9.4).

## Results

### Group Comparisons

The NC and the LMCI groups did not differ in age, years of education, occupational status, hippocampal GM MD and premorbid intelligence (Table 1). We found a slightly higher proportion of men in the LMCI group compared to the NC group (non-significant). As expected, the MMSE was lower in the LMCI group than in the NC group. Compared to controls, hippocampal volume was reduced in the LMCI group. Also, the proportion of amyloid positive participants was higher in the LMCI group than in the NC group.

**TABLE 1:** Sample characteristics and group comparisons.

**Group**	**NC**	**LMCI**	**Group comparisons**
No. (women)	52 (27)	38 (13)	No. of women: LMCI < NC^†^
Age, *M* (SD)	73.6 (5.9)	75.1 (7.6)	n.s.
Amyloid positivity (Amyloid negative/Amyloid positive)	37 / 15	12 / 26	No. of Amyloid positive participants: LMCI > NC*
Years of education, M (SD)	16.4 (2.8)	16.5 (2.7)	n.s.
MMSE, *M* (SD)	28.7 (1.5)	27.7 (1.8)	NC > LMCI*
Hippocampal Volume in ml, *M* (SD)	7.9 (0.9)	7.2 (0.9)	NC > LMCI*
Hippocampal GM MD *10^–3^ mm^2^/s, *M* (SD)	1.18 (0.11)	1.21 (0.08)	n.s.
ANART, *M* (SD)	10.3 (8.3)	13.5 (10.8)	n.s.
Occupational Status (1/2/3)^1^	17/22/13	12/14/12	n.s.

^1^1, professional or managerial occupations / 2, skilled occupations / 3, partly skilled or unskilled occupations. *p < 0.05, ^†^*p* < 0.1. Abbreviations: ANART, American National Adult Reading Test; LMCI, late mild cognitive impairment; M, mean; MMSE, mini mental status examination; n.s., not significant; NC, normal cognition; SD, standard deviation.

### Association of Hippocampal GM MD and CR

We did not find an overall effect of CR on hippocampal GM MD in either group (LMCI and NC) (Table 2).

**TABLE 2:** Linear model regressing hippocampal GM MD on the CR score, adjusted for age, gender composite memory score and study phase (LMCI).

	**LMCI (*N* = 38)**			**NC (*N* = 52)**		
	***β***	***T***	***p***	***β***	***T***	***p***
**Main effects**						
CR score	0.03	0.17	0.86	0.04	0.29	0.77
**Covariates**						
Age	0.43	3.01	0.005	0.41	3.27	0.002
Gender	0.05	0.34	0.74	0.24	1.90	0.06
Composite memory score	–0.08	0.51	0.62	–0.21	–1.56	0.13
Study phase	–0.52	–3.18	0.003	–	–	–
*R*^2^	0.39			0.42		
Power	0.96			>0.99		

*Standardized regression weights (*β)* resulting from linear regression. The coefficient of determination (*R*^2^) is not adjusted. Abbreviations: CR, cognitive reserve; GM MD, gray matter mean diffusivity; LMCI, late mild cognitive impairment; NC, normal cognition.*

### Effects of Cognitive Reserve and Amyloid Status on Hippocampal GM MD

In this analysis, we added amyloid status as a dichotomous predictor of hippocampal GM MD. Again, there was no significant main effect of CR on hippocampal GM MD and no interaction effect (Table 3). However, we found a positive effect of amyloid positivity on hippocampal GM MD in the LMCI subgroup, indicating higher hippocampal MD in amyloid positive LMCI participants.

**TABLE 3:** Linear model regressing hippocampal MD on cognitive reserve and amyloid status, adjusted for age, gender, composite memory score and study phase (LMCI).

	**LMCI (*N* = 38)**			**NC (*N* = 52)**		
	***β***	***T***	***p***	***β***	***T***	***p***
**Main effects**						
CR score	0.05	0.18	0.89	0.03	0.20	0.84
Amyloid status	0.33	2.13	0.04	–0.06	–0.49	0.62
Amyloid status* CR	–0.10	–0.42	0.68	–0.003	–0.02	0.96
**Covariates**						
Age	0.45	3.24	0.003	0.42	3.22	0.002
Gender	0.09	0.63	0.53	0.22	1.64	0.11
Composite memory score	0.05	0.29	0.77	–0.22	–1.59	0.12
Study phase	–0.45	–2.8	0.009	–	–	–
*R*^2^	0.47			0.42		
Power	0.95			>0.99		

*Standardized regression weights (*β)* resulting from linear regression. The coefficient of determination (*R*^2^) is not adjusted. Abbreviations: CR, cognitive reserve; GM MD, gray matter mean diffusivity; LMCI, late mild cognitive impairment; NC, normal cognition.*

### Association of Hippocampal Volume and CR

There was no significant main effect or interaction of CR or amyloid status on hippocampal volume (all *p*-values > 0.05). We found a trend toward a negative effect of amyloid status, indicating higher hippocampal volume in LMCI participants with low amyloid burden (Table 4).

**TABLE 4:** Linear model regressing hippocampal volume on cognitive reserve and amyloid status, adjusted for age, gender, composite memory score and study phase (LMCI).

	**LMCI (*N* = 38)**			**NC (*N* = 52)**		
	**β**	***T***	***p***	**β**	***T***	***p***
**Main effects**						
CR score	–0.02	–0.06	0.95	0.01	0.10	0.92
Amyloid status	–0.2.8	–1.91	0.07	–0.20	–1.62	0.11
Amyloid status * CR	0.03	0.11	0.91	0.07	0.47	0.64
**Covariates**						
Age	–0.42	–3.12	0.004	–0.35	–2.72	0.01
Gender	–0.35	–2.66	0.01	–0.42	–3.09	0.003
Composite memory score	0.21	1.35	0.19	0.11	0.79	0.44
Study phase	0.15	0.94	0.35	–	–	–
*R*^2^	0.51			0.44		
Power	>0.99			>0.99		

*Standardized regression weights (*β)* resulting from linear regression. The coefficient of determination (*R*^2^) is not adjusted. Abbreviations: CR, cognitive reserve; GM MD, gray matter mean diffusivity; LMCI, late mild cognitive impairment; NC, normal cognition.*

## Discussion

The aims of the current study were (1) to explore the association of hippocampal microstructural integrity and CR that has been found in middle-aged adults (Piras et al., 2011) in older participants without dementia and (2) to examine the differential effects of CR on hippocampal GM MD in older people without dementia with and without amyloid burden. We found that hippocampal GM MD was not related to CR in older adults without dementia. Also, no interaction of amyloid status and CR was found. Piras et al. (2011) found a negative association of years of education and hippocampal MD in middle-aged healthy adults. We could not replicate this finding in older people without dementia. The present study differs from the study by Piras et al. (2011) in several ways. First, while Piras et al. (2011) chose middle-aged healthy adults, we studied older, non-demented participants because CR takes effect in this age group. One could argue that our sample may have been too old to detect an effect of CR, which is closely related to school education. However, the effect of education on the risk of dementia and on dementia pathology at a more advanced age has been shown in many studies (Mortimer et al., 2003; Valenzuela and Sachdev, 2006; Meng and D’Arcy, 2012) and, crucially, is the key finding supporting the CR hypothesis (Stern, 1994, 2009). Second, we corrected for PVE when estimating hippocampal GM MD. By not using any partial volume correction, Piras et al. (2011) potentially overestimated MD in the older participants of their sample (Henf et al., 2018). However, this should not essentially change the patterns of results in middle-aged participants, because PVE are more pronounced in older than in younger people (Jeon et al., 2012). Additionally, the choice of covariates in our study differs from Piras et al. (2011), who used age as the sole nuisance variable. However, both gender and cognitive performance need to be included when analyzing the association of CR and measure of regional brain integrity (Arenaza-Urquijo et al., 2015). Especially memory performance seems to be related to CR (Opdebeeck et al., 2016). Therefore, when omitting memory performance as a covariate in these analyses, the resulting associations of CR and neuroimaging data may reflect differences in cognitive performance instead of differences in CR. Moreover, both subgroups of our sample had more years of education than the sample by Piras et al. (2011). This results from the high education of the ADNI sample in general^4^. The high education limits the generalizability of our findings to the general population. What is more, the choice of CR proxy differed, as Piras et al. (2011) used years of education as the proxy variable and we used a composite CR score. However, our composite score resulted from a factor analysis that also included years of education. In fact, we observed a high correlation between years of education and the composite CR score (Supplementary Material and Supplementary Table S1). When repeating the regression analysis with years of education as the sole CR proxy, the results did not change substantially (Supplementary Material and Supplementary Table S2). In addition to that, the sample size of Piras et al. (2011) was considerably larger than our sample size, which might suggest a potential lack of power of our analyses. However, as can be seen in the section “Results” (Tables 2–4), all power values were within a range of 0.95 to >0.99. Therefore, we conclude that both samples were sufficiently large to detect a potential effect of CR.

Our study also aimed at determining the differential effects of CR on hippocampal microstructural integrity according to cognitive and amyloid status. Previous studies on CR proposed that higher CR leads to decreased neuropathology (“neuroprotective effect”) in healthy individuals (Arenaza-Urquijo et al., 2015). In individuals with existing neuropathology, those with a higher CR may tolerate a higher amount of pathology, leading to the inverse association of higher CR being associated with increased neuropathology (“compensatory effect”). Our results do not conform to this model suggested by Arenaza-Urquijo et al. (2015). In the overall statistical models, none of the main effects or interaction terms reached significance. Possibly, hippocampal GM MD is not sufficiently sensitive to neurodegeneration. However, using hippocampal volume as a criterion in the regression analyses, the results did not essentially change. What is more, the CR score in the present study was composed of a factor representing three CR proxies and resulted from a factor analytic approach. Compared to previous studies using this approach, the percentage of variance explained by our score was relatively low (55%), indicating lower intercorrelations of the original variables (years of education, occupation and ANART). The ANART may be sensitive not only to *premorbid*, but also to *current* cognitive performance (Lowe and Rogers, 2011). In our sample, we found a numerically higher number of errors in the ANART in the LMCI sample compared to the NC sample. Albeit not statistically significant, this difference may also result from reduced memory performance in the LMCI sample instead of premorbid IQ. Thus, the composite CR score in the LMCI sample may be confounded by cognitive impairment. This may have limited its correlation with the other two CR proxies used in this study (years of education and occupation).

Several limitations need to be considered in the present study. First, the acquisition delay between the PET and the MRI scans was variable in the present sample (*M* = 73 days; SD = 136), even though binary amyloid positivity remained stable during the study period in those participants with the longest acquisition delays (>100 days). Unfortunately, information on CSF biomarkers and/or Tau PET was not available for our sample. These biomarkers would have helped to more accurately quantify neuropathology in the cognitively normal participants. Also, some of the participants did not receive a diffusion weighted scan at baseline but only in the course of the study. Thus, slight retest effects may have influenced the memory score (Crane et al., 2012). However, exclusion of non-baseline data did not substantially change the results (data not shown). Another limitation is the *post hoc* quantification of the occupational status. A more differentiated assessment such as the lifetime of experiences questionnaire (Valenzuela and Sachdev, 2007) or a longer version of the National Statistics Socio-economic Classification (Chandola and Jenkinson, 2000) would have been desirable. However, the information available in ADNI was not detailed enough to determine more nuanced scales of occupational attainment. We took this limitation into account by calculating a composite CR score containing less method-specific variance than the single CR proxies available in the ADNI dataset (years of education/occupation/ANART).

## Conclusion

In this study, we aimed at determining the role of hippocampal microstructural integrity in the neuronal mechanisms of CR. We did not find an association of CR and hippocampal GM MD in healthy participants and in LMCI patients. Also, we did not find amyloid load to modulate the association of hippocampal GM MD and CR. On a neuronal level, we therefore assume that there is no association of beginning neurodegeneration, as indicated by an increase of hippocampal MD, and CR in older people. Thus, CR does not seem to take effect by preventing or compensating early hippocampal damage in older healthy people. More research is needed to determine the exact role of hippocampal MD in the interplay of age, AD pathology and CR. The data from the ADNI 3 study may help to address these questions in future studies.

## Data Availability Statement

The datasets generated for this study are available on request to the corresponding author.

## Ethics Statement

Ethical approval for data collection and sharing was given by the institutional review boards of the participating institutions in the ADNI. The patients/participants provided their written informed consent to participate in this study.

## Author Contributions

JK and ST contributed to the design of the study. JK performed the statistical analysis and wrote the first draft of the manuscript. All authors contributed to the interpretation of the data and to manuscript revision, and read and approved the submitted version.

## Conflict of Interest

The authors declare that the research was conducted in the absence of any commercial or financial relationships that could be construed as a potential conflict of interest.

## Footnotes

1 adni.loni.usc.edu

2 http://adni.loni.usc.edu/methods/pet-analysis

3 http://www.fil.ion.ucl.ac.uk/spm/

4 https://adni.loni.usc.edu/wp-content/uploads/2012/08/ADNI_Enroll_Demographics.pdf

## Abbreviations

A β−: amyloid negative
A β +: amyloid positive
AD: Alzheimer’s disease
ANART: American National Adult Reading Test
CR: cognitive reserve
CSF: cerebrospinal fluid
DTI: diffusion tensor imaging
GM: gray matter
LMCI: late mild cognitive impairment
MCI: mild cognitive impairment
MRI: magnet resonance imaging
NC: normal cognition
PET: positron emission tomography
PVE: partial volume effects.
