# Supplementary material for: Cognitive Reserve Is Not Associated With Hippocampal Microstructure in Older Adults Without Dementia
Source: Front Aging Neurosci. 2020 Jan 23;11:380. doi: 10.3389/fnagi.2019.00380 (PMC7081775; doi:10.3389/fnagi.2019.00380)
Supplement: Supplementary file 1 [file Data_Sheet_1.PDF]

Supplementary Material

1. Data distributions

1.1 Histograms of the cognitive reserve score

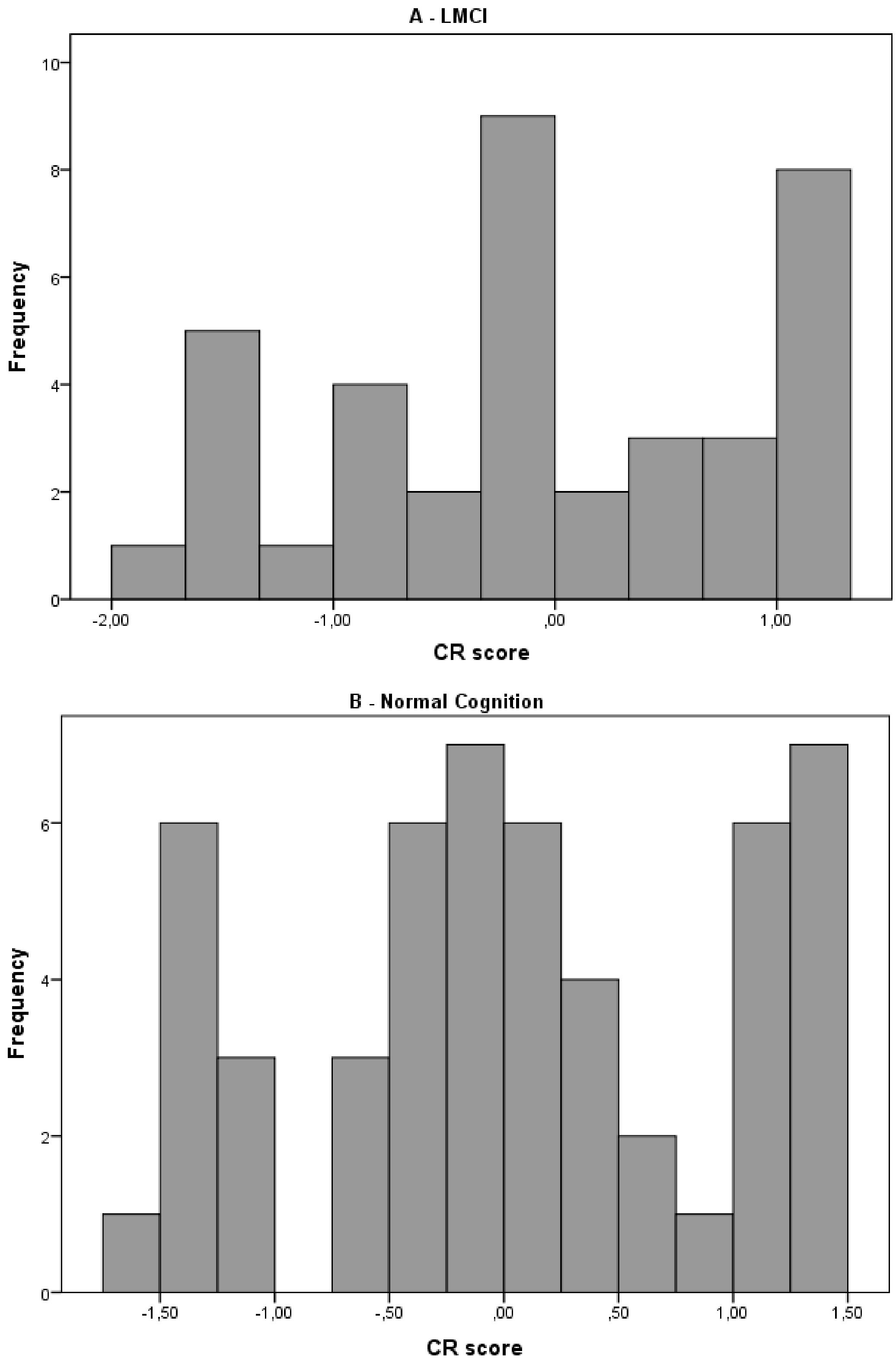

Figure S1. Histograms of the cognitive reserve score used in the present study. A – LMCI sample; B – Normal Cognition sample.
Abbreviations: CR, cognitive reserve.

1.2 Scatterplots of hippocampal mean diffusivity

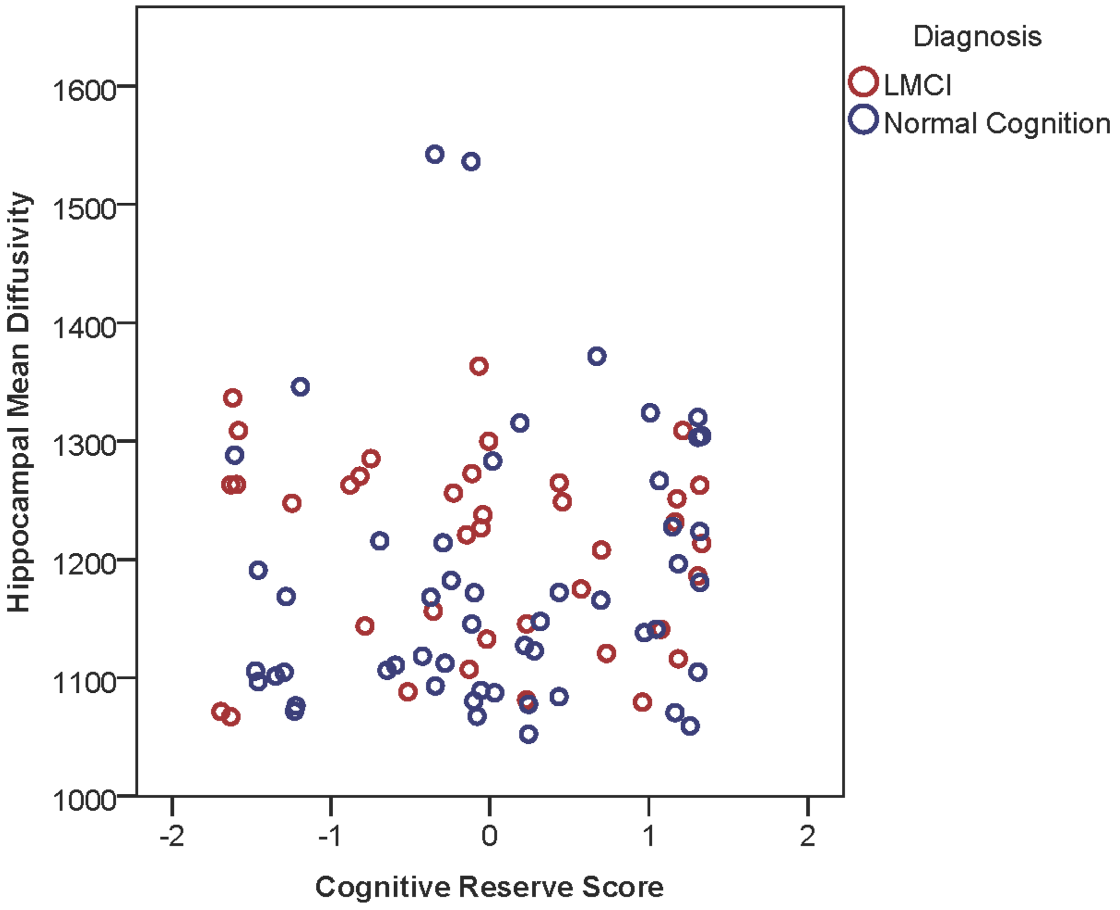

Figure S2. Scatterplot of the cognitive reserve score and hippocampal gray matter mean diffusivity (10^{-6} mm²/s) in the late mild cognitive impairment (LMCI) and the normal cognition group.

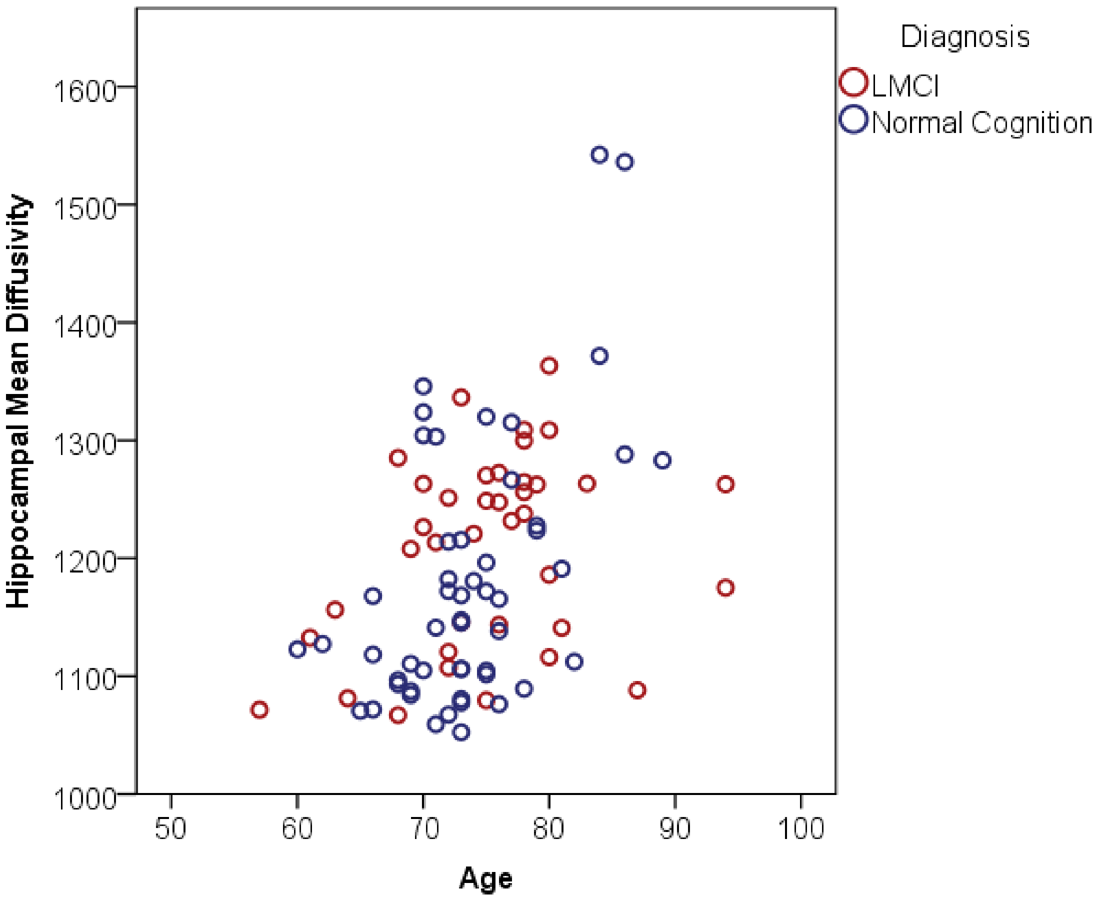

Figure S3. Distribution of age and hippocampal mean diffusivity (10^{-6} mm²/s) according to diagnostic groups.
Abbreviation: LMCI, late mild cognitive impairment.

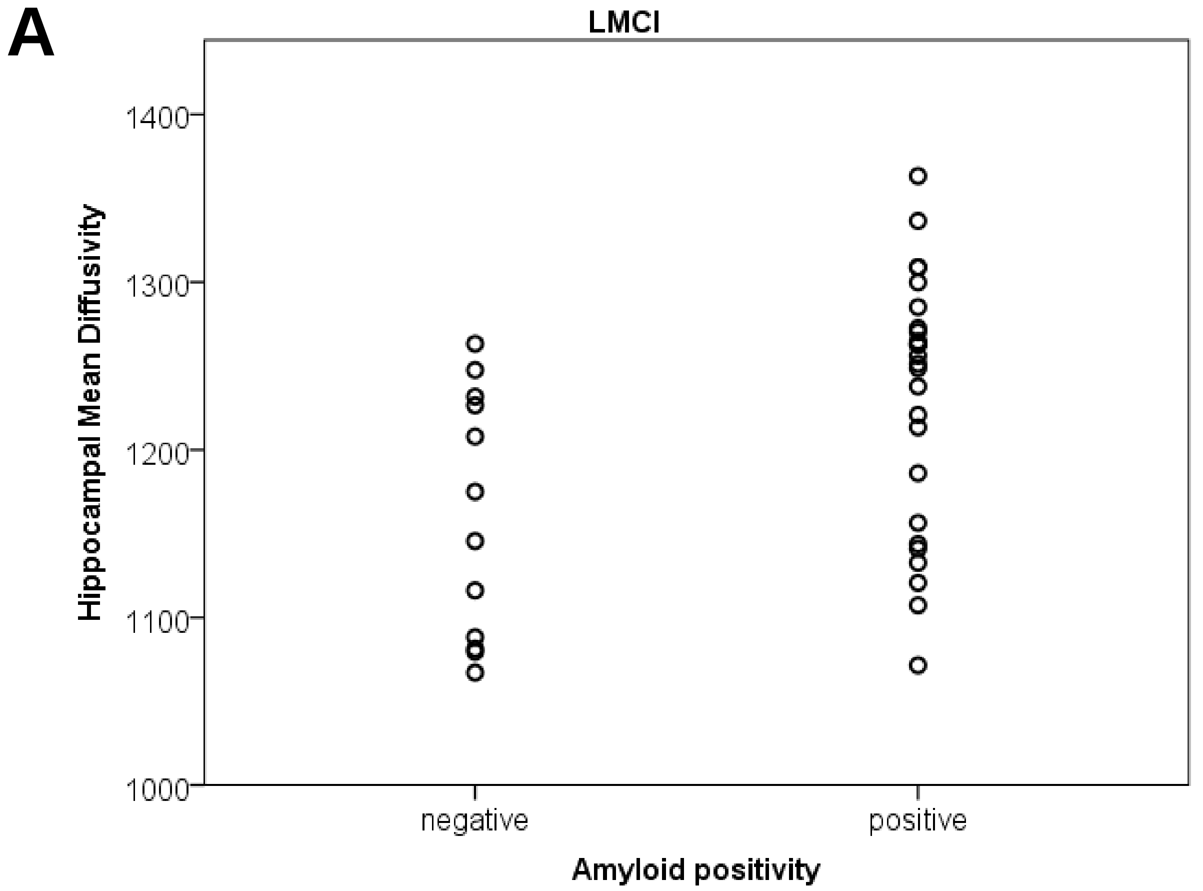

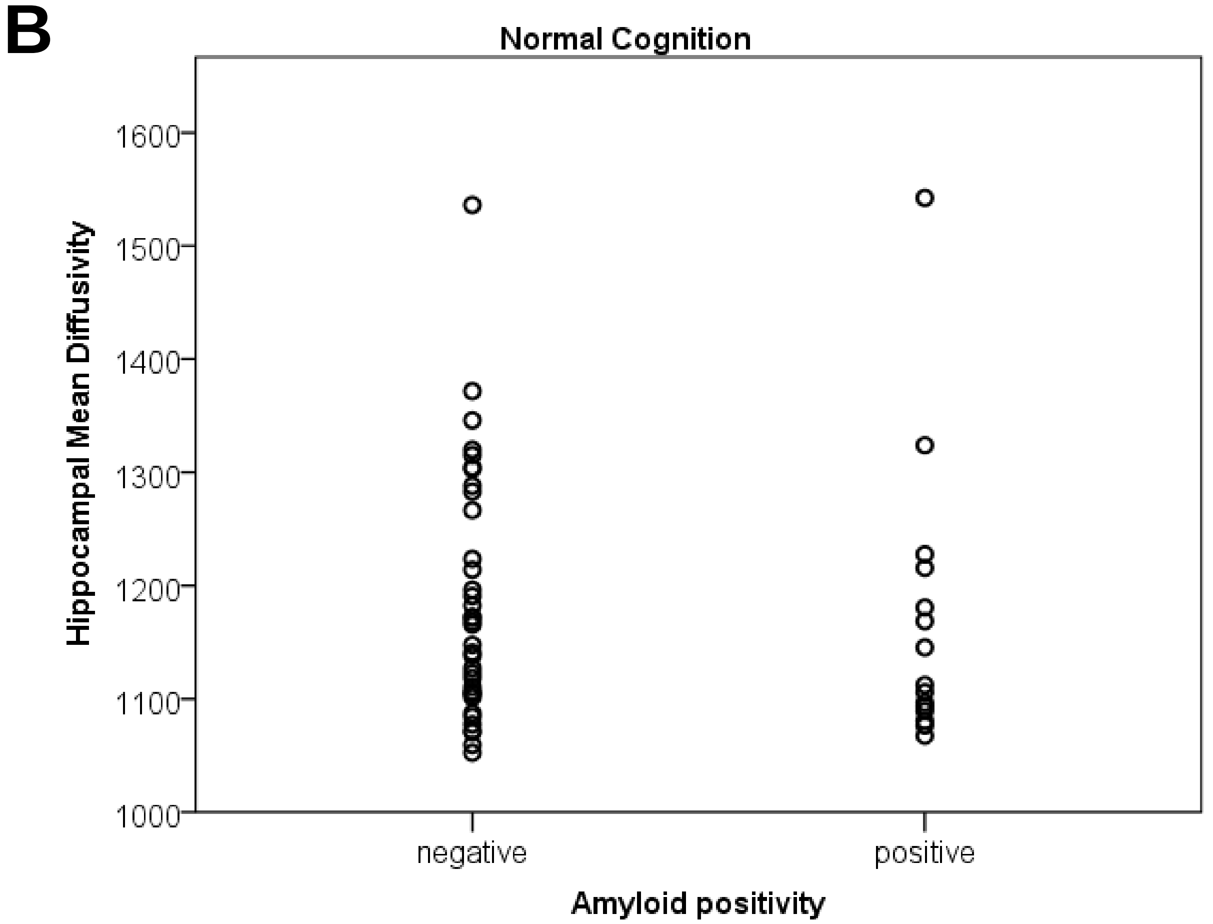

Figure S4. Distribution of amyloid positivity and hippocampal mean diffusivity (10^{-6} mm²/s). A - late mild cognitive impairment (LMCI), B - Normal Cognition.

1.3 Hippocampal Gray Matter Volume

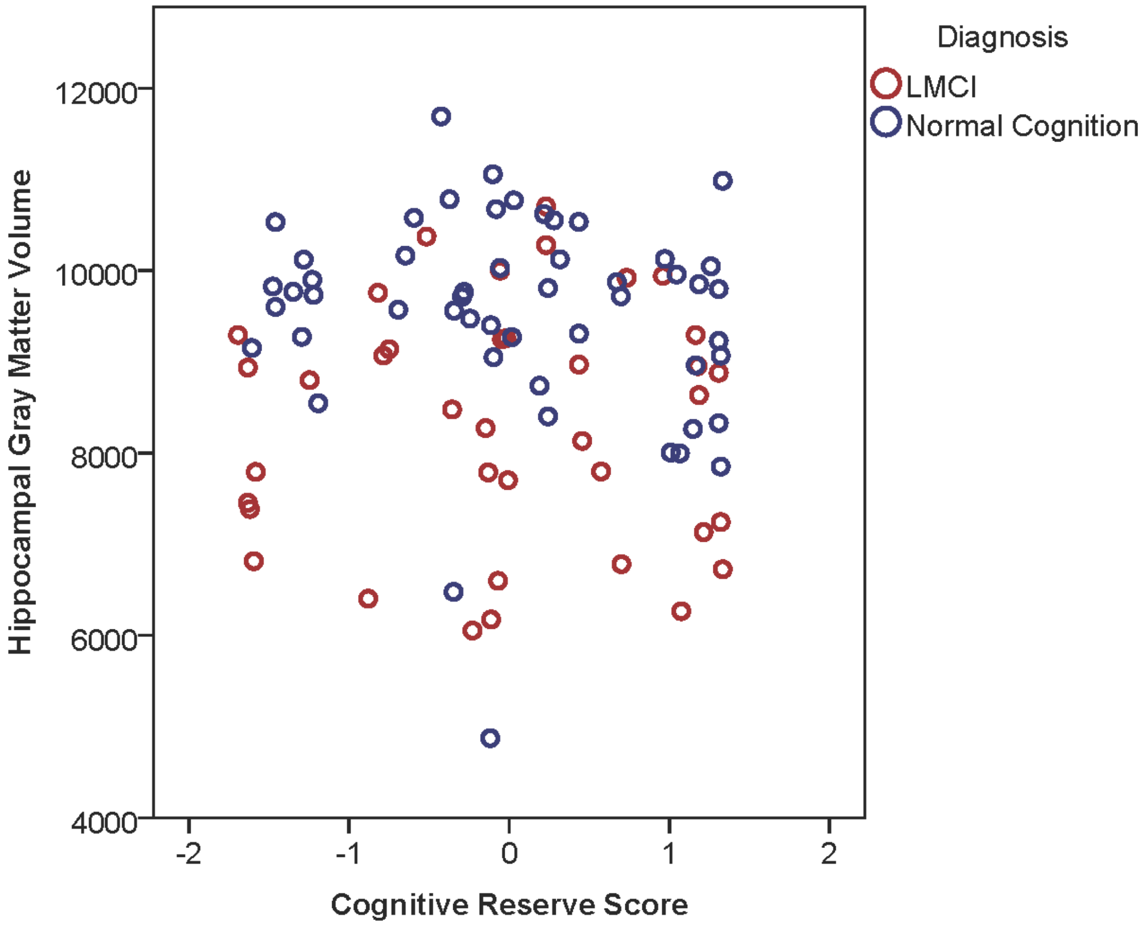

Figure S5. Scatterplot of the cognitive reserve score and hippocampal gray matter volume (mm³) in the late mild cognitive impairment (LMCI) and the normal cognition group.

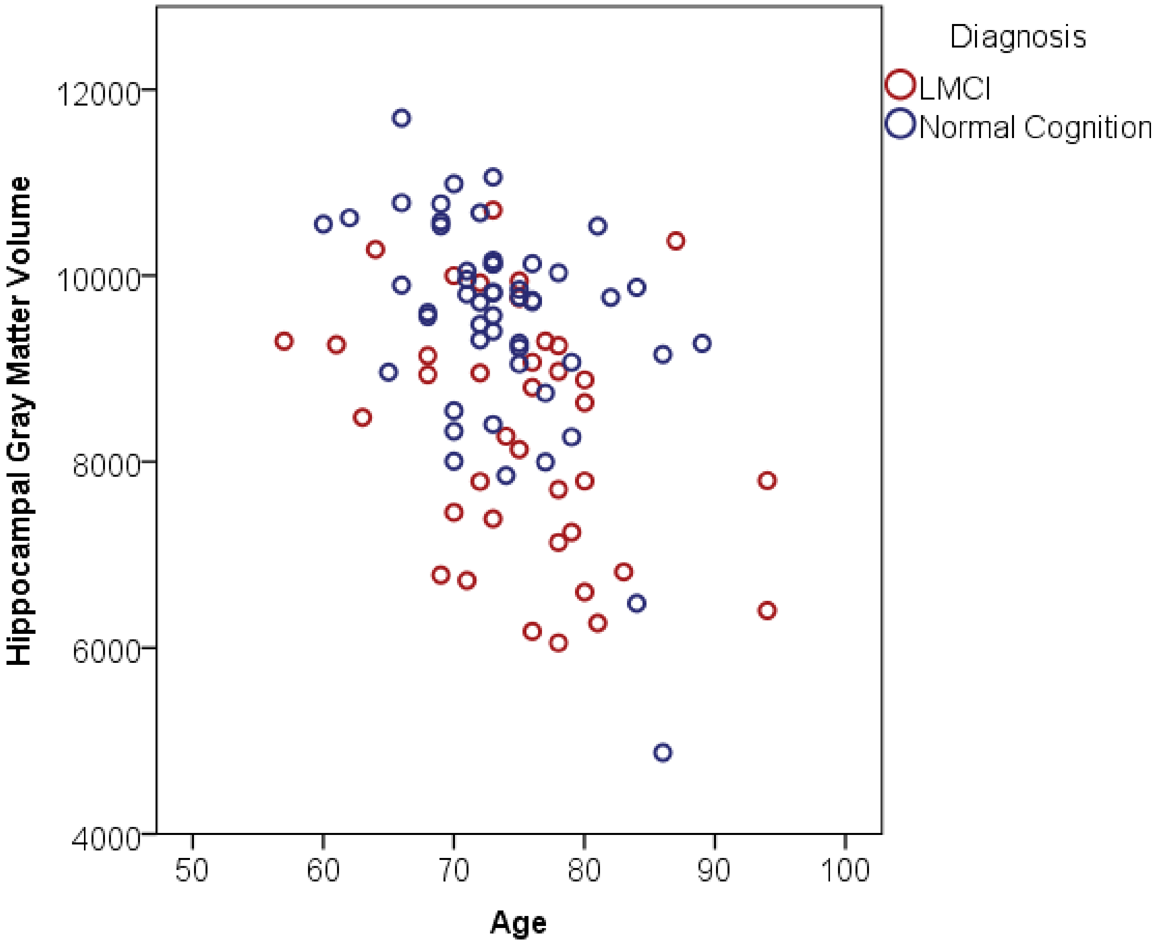

Figure S6. Distribution of age and hippocampal gray matter volume (mm³) according to diagnostic groups.
Abbreviation: LMCI, late mild cognitive impairment.

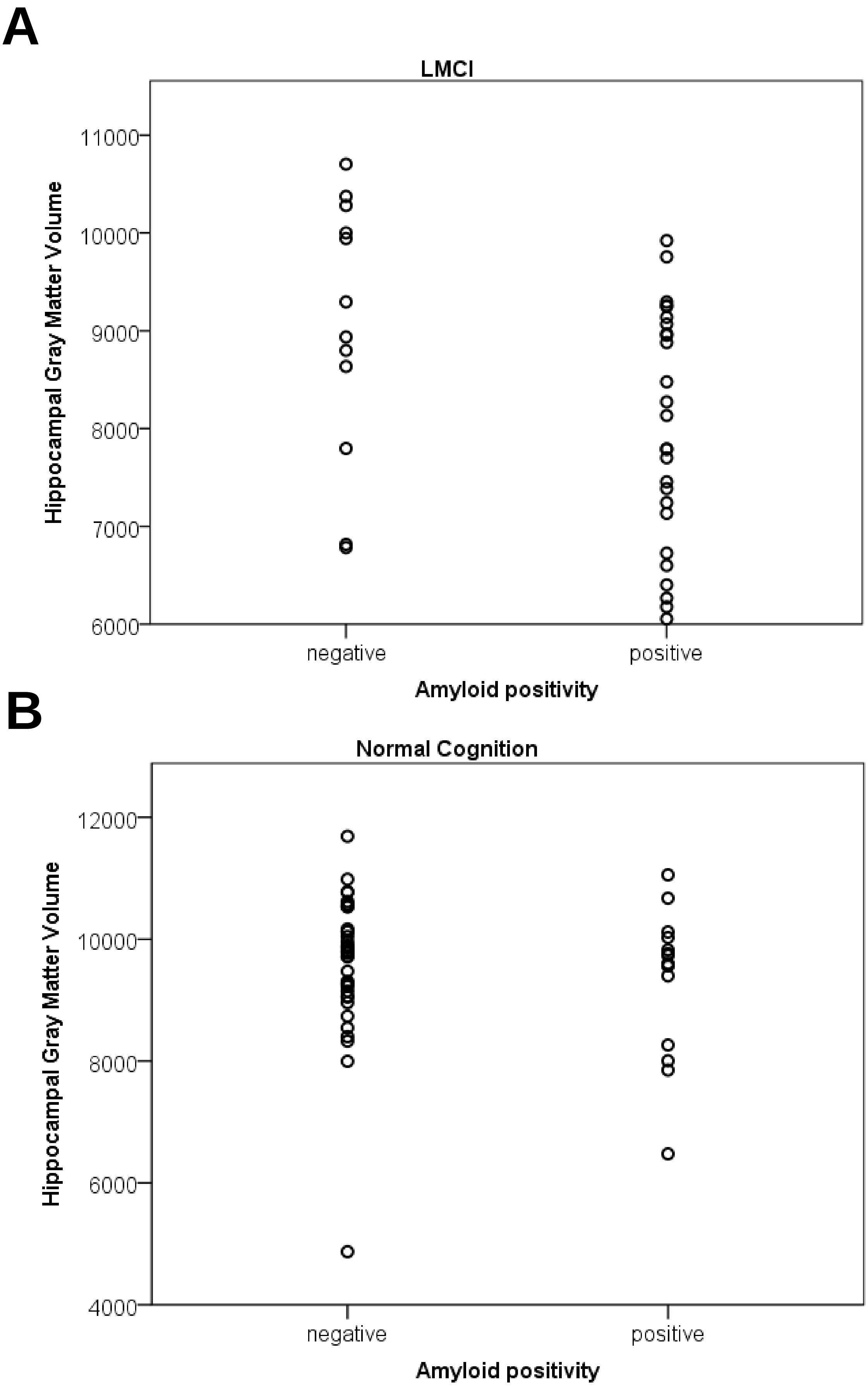

Figure S7. Distribution of amyloid positivity and hippocampal mean gray matter volume (mm^3). A - late mild cognitive impairment (LMCI), B - Normal Cognition.

2. Results of the principal component analysis in R

Table S1. Results of the principal component analysis in R.

	Factor loading	Communality
ANART Errors	-0.52	0.27
Years of education	0.82	0.67
Occupation	-0.85	0.73

Abbreviations: ANART, American National Adult Reading Test.

Eigenvalue: 1.66

Proportion of explained variance: 0.55

3. Results using years of education as cognitive reserve proxy

Table S2. Linear model regressing hippocampal GM MD on years of education, adjusted for age, gender composite memory score and study phase (LMCI).

		LMCI (*N*=38)			NC (*N*=52)		
		β	*T*	*p*	*β*	*T*	*p*
Main effects	Years of education	.11	.74	.47	.12	.95	.35
Covariates	**Age**	**.43**	**3.02**	**<.01**	**.42**	**3.4**	**.001**
	Gender	.03	.18	.86	.19	1.44	.16
	Composite memory score	-.09	-.55	.59	-.20	-1.57	.12
	Study phase	**-.54**	**-3.33**	**<.01**	-	-	-
Overall model fit	R^2	.39			.43		
Power		.96			<.99		

Standardized regression weights (*β*) resulting from linear regression. The coefficient of determination (R^2) is not adjusted.
Abbreviations: CR, cognitive reserve; GM MD, grey matter mean diffusivity; LMCI, late mild cognitive impairment; NC, normal cognition.

Table S3. Linear model regressing hippocampal MD on years of education and amyloid status, adjusted for age, gender, composite memory score and study phase (LMCI).

		LMCI (*N*=38)			NC (*N*=52)		
		β	*T*	*p*	*β*	*T*	*p*
Main effects	Years of education	.17	.82	.42	.13	.92	.36
	**Amyloid status**	**.30**	**1.99**	**.056**	-.06	-.50	.62
	**Amyloid status*** CR	.18	.89	.38	.03	.21	.84
Covariates	**Age**	**.45**	**3.3**	**.003**	**.43**	**.34**	**.002**
	Gender	.07	.47	.64	.18	1.26	.21
	Composite memory score	.05	.32	.75	-.22	-1.6	.11
	**Study phase**	**-.48**	**-2.96**	**.006**	-	-	-
Overall model fit	R^2	.48			.43		
Power		.99			<.99		

Standardized regression weights (*β*) resulting from linear regression. The coefficient of determination (R^2) is not adjusted.

Abbreviations: CR, cognitive reserve; GM MD, grey matter mean diffusivity; LMCI, late mild cognitive impairment; NC, normal cognition.
